# Assessment of Local Public Health Workers' Willingness to Respond to Pandemic Influenza through Application of the Extended Parallel Process Model

**DOI:** 10.1371/journal.pone.0006365

**Published:** 2009-07-24

**Authors:** Daniel J. Barnett, Ran D. Balicer, Carol B. Thompson, J. Douglas Storey, Saad B. Omer, Natalie L. Semon, Steve Bayer, Lorraine V. Cheek, Kerry W. Gateley, Kathryn M. Lanza, Jane A. Norbin, Catherine C. Slemp, Jonathan M. Links

**Affiliations:** 1 Johns Hopkins Center for Public Health Preparedness, Baltimore, Maryland, United States of America; 2 Johns Hopkins Preparedness and Emergency Response Research Center, Baltimore, Maryland, United States of America; 3 Department of Environmental Health Sciences, Johns Hopkins Bloomberg School of Public Health, Baltimore, Maryland, United States of America; 4 Department of Epidemiology, Faculty of Health Sciences, Ben-Gurion University of the Negev, Beer-Sheva, Israel; 5 Department of Biostatistics, Johns Hopkins Bloomberg School of Public Health, Baltimore, Maryland, United States of America; 6 Johns Hopkins Bloomberg School of Public Health/Center for Communication Programs, Baltimore, Maryland, United States of America; 7 Department of Health, Behavior and Society, Johns Hopkins Bloomberg School of Public Health, Baltimore, Maryland, United States of America; 8 Hubert Department of Global Health, Emory University, Rollins School of Public Health, Atlanta, Georgia, United States of America; 9 Mid-Ohio Valley Region, Parkersburg, West Virginia, United States of America; 10 Preble County General Health District, Wright State University Masters of Public Health, Eaton, Ohio, United States of America; 11 Kanawha-Charleston Health Department, Charleston, West Virginia, United States of America; 12 Summit County Health District, Stow, Ohio, United States of America; 13 St. Paul-Ramsey County Department of Public Health, St. Paul, Minnesota, United States of America; 14 Bureau for Public Health, West Virginia Department of Health and Human Resources, Charleston, West Virginia, United States of America; Fred Hutchinson Cancer Research Center, United States of America

## Abstract

**Background:**

Local public health agencies play a central role in response to an influenza pandemic, and understanding the willingness of their employees to report to work is therefore a critically relevant concern for pandemic influenza planning efforts. Witte's Extended Parallel Process Model (EPPM) has been found useful for understanding adaptive behavior in the face of unknown risk, and thus offers a framework for examining scenario-specific willingness to respond among local public health workers. We thus aim to use the EPPM as a lens for examining the influences of perceived threat and efficacy on local public health workers' response willingness to pandemic influenza.

**Methodology/Principal Findings:**

We administered an online, EPPM-based survey about attitudes/beliefs toward emergency response (*Johns Hopkins∼Public Health Infrastructure Response Survey Tool*), to local public health employees in three states between November 2006 – December 2007. A total of 1835 responses were collected for an overall response rate of 83%. With some regional variation, overall 16% of the workers in 2006-7 were not willing to “respond to a pandemic flu emergency regardless of its severity”. Local health department employees with a perception of high threat and high efficacy – i.e., those fitting a ‘concerned and confident’ profile in the EPPM analysis – had the highest declared rates of willingness to respond to an influenza pandemic if required by their agency, which was 31.7 times higher than those fitting a ‘low threat/low efficacy’ EPPM profile.

**Conclusions/Significance:**

In the context of pandemic influenza planning, the EPPM provides a useful framework to inform nuanced understanding of baseline levels of – and gaps in – local public health workers' response willingness. Within local health departments, ‘concerned and confident’ employees are most likely to be willing to respond. This finding may allow public health agencies to design, implement, and evaluate training programs focused on emergency response attitudes in health departments.

## Introduction

The anticipated worldwide morbidity, mortality, and social disruption from an influenza pandemic [1] require detailed and tested approaches to staffing and resource allocation in public health systems [2]. The willingness of health responders to report to duty during an influenza pandemic is a highly salient concern given the “inevitable”nature of this threat [3] and its associated challenges. Scant margin exists in the nation's public health system for local health department workers – the backbone of public health system readiness – to “opt out” of response duties, given limitations of health system surge capacity [4], public health personnel shortages [5], and continued steep learning curves associated with relatively new 24/7 response expectations for health department employees.

The unwillingness of some health workers to place themselves at risk of exposure to emerging infectious diseases was observed during the 2003 SARS epidemic and the early years of the HIV/AIDS epidemic [6]. In the aftermath of the terror attacks of September 11, 2001 and the ensuing anthrax bioterrorism attacks, a growing body of research literature has examined willingness to respond to large-scale emergencies among a variety of health-related cohorts [7]–[14]. Despite the evidence for fundamental distinctions between ability and willingness to respond [7], [13], there remains a gap in the public health preparedness literature on training approaches that explicitly address response *willingness* (attitude) as a discrete outcome. Based on the principle that “all disasters begin locally”, these observations underscore a fundamental need to understand root causes of local public health workers' barriers to response willingness, as a basis for identifying and addressing public health response system gaps in this domain.

A variety of risk perception theories have been suggested and may help to identify barriers to health personnel adopting an emergency responder role. One prominent model conceptualizes risk perception as the sum of “hazard” and “outrage”, where *hazard* is a product of risk magnitude and probability, and *outrage* is a function of other peripheral influences independent of the actual risk, such as perceived authority, trust, and situational control [15].

Among the public health workforce, recent applications of this “Risk = Hazard+Outrage” model have uncovered a variety of potential peripheral risk perception influences on health department workers' response willingness apart from the actual hazard [16]. For example, in a 2005 pilot study conducted in three local health departments in Maryland, we found that a health department employee's individual perceived level of importance in their agency's response efforts was a particularly strong peripheral influence on response willingness toward an influenza pandemic [8].

The cumulative evidence from these studies suggests that willingness to respond is multidimensional. Specifically, its dimensions appear to include: 1) *perceived threat*, as evidenced by findings of scenario-specific response willingness rates; and 2) *perceived efficacy*, as highlighted by the powerful influences of response efficacy (“My response makes a difference”) and self-efficacy (“I can do what is expected of me as a responder”). Further, preparedness training for public health workers is a form of risk communication in itself, intended to build health department workers' efficacy in the face of a variety of hazards. To build a public health workforce that is not only able to respond, but also willing to do so, the above observations suggest the need for a unifying paradigm that can address both the threat and efficacy dimensions of willingness to respond. To date, the research literature on public health emergency response willingness has lacked such a paradigm.

**Figure 1:**
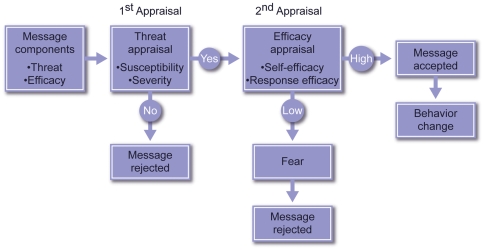
Extended Parallel Process Model. Witte's Extended Parallel Process Model (EPPM) describes how people, when faced with a potential hazard, will sequentially appraise the threat and efficacy content of related health and safety protection messages, and will respond accordingly. The first appraisal is for *threat* [threat appraisal]. The threat appraisal has two components: *severity* and *susceptibility*. If, in the threat appraisal, the message recipient personally perceives the hazard to be of negligible consequence (low severity) or improbable (low susceptibility), any related message content encouraging a desired protection-oriented response or behavior will be rejected. If, however, the message passes the threat appraisal, the message recipient will next process the message's content for *efficacy* [efficacy appraisal]. The efficacy appraisal contains two components: *self-efficacy* and *response efficacy*. If the message recipient does not find the message's targeted behavior to be achievable (low self-efficacy) or efficacious (low response efficacy), the message recipient will engage in undesirable responses such as denial and avoidance in order to manage fear (described as “fear control” in the EPPM); this will be accompanied by message rejection. If, however, the efficacy appraisal is also passed, message acceptance will result, leading to adoption of the message's intended protective behavior change outcomes by taking desirable steps to minimize personal risk against the actual hazard (described as “danger control” in the EPPM).

The Extended Parallel Process Model (EPPM) [Figure 1] has been found to be useful for understanding adaptive behavior in the face of unknown risk [17]. First proposed by Witte in the early 1990s [18], the EPPM represents an integration and expansion of previous psychosocial models of “fear appeal.” The model focuses on messages that are received by both individuals and collectively by groups. Importantly, while the model was first developed to explain individual behavior [18], it has since been directly applied to the analysis of collective behavior [19].

According to the EPPM, in order to be effective, messages must contain two parts: threat and efficacy. Message receivers sequentially perform two appraisals, first of threat and then of efficacy. The first portion of the message must convincingly transmit the existence of a threat, leading to concern on the part of the message receiver(s). The second portion of the message must convincingly transmit the existence of efficacious interventions/mitigations, especially those that are self-efficacious (i.e., able to be performed by the message receivers), leading to confidence. According to this model, the threat and efficacy components must be accepted by the message receivers to achieve the desired behavior or practice (at both individual and collective levels); this is termed “danger control” per the EPPM. If the threat portion is not accepted, the message is rejected. If the threat portion is accepted, but the efficacy portion is not, the acceptance of the threat portion triggers fear, which the message receivers attempt to manage (by rejecting the entire message); such a reaction is referred to as “fear control” per the EPPM.

Through the design and administration of an EPPM-centered survey of local health department personnel in three states, we aim to examine the relative influences of perceived threat and efficacy on public health workers' response willingness to pandemic influenza.

## Methods

### Ethics Statement

Research ethics approval for the *Johns Hopkins∼Public Health Infrastructure Response Survey Tool (JH∼PHIRST)* survey and its administration was received from the Johns Hopkins Bloomberg School of Public Health Institutional Review Board (JHSPH IRB) (exempt status # 45 CFR 46.101 (b) (2)). Per JHSPH IRB approval, written consent was not obtained, as the research presented no more than minimal risk to subjects and involved no procedures for which written consent is normally required. The JHSPH IRB-approved study materials included a written disclosure describing the study and emphasizing voluntary participation; verbal consent was not requested or required by JHSPH IRB for this approved study.

### Survey Instrument

The *JH∼PHIRST* is an anonymous online survey instrument consisting of demographic and attitude/belief sections focusing on health department workers' attitudes and beliefs toward public health emergency response. The demographic information includes gender, highest education level, role in an emergency response, responsibility for a family member, and categories of age, professional classification, years in present organization, and years in profession. For each of four emergency scenarios (weather-related emergency, pandemic influenza, ‘dirty bomb’ radiological terrorism event, and inhalational anthrax bioterrorism event) the same 20 attitudes and beliefs were presented for level of agreement along with two open-ended questions. Responses to the attitude and belief questions were based on a 10-point Likert scale with a response of ‘1’ indicating strong agreement with the question and a response of ‘10’ indicating strong disagreement with the question. Respondents could also indicate “don't know”.

The online *JH∼PHIRST* instrument used in the current study evolved from an earlier, paper-based pilot survey instrument that we implemented in three Maryland local health departments in 2005 to assess the willingness of these employees to respond to an influenza pandemic [8]. Of note, while this earlier pilot version did incorporate some aspects of risk perception, it did not use the threat and efficacy measures from EPPM that have become commonplace in risk communication studies over the past decade. We incorporated the EPPM content to generate the online *JH∼PHIRST* instrument based on a series of relevant observations from the 2005 Maryland-based pilot study – namely, that local public health workers' willingness to respond in a pandemic flu scenario was hampered by uncertainties, fears and lack of confidence [8] that are reflected in the standard batteries of questions used by EPPM studies.

The online *JH∼PHIRST* survey's EPPM-based threat and efficacy measures have been widely validated by numerous studies in multiple countries, cultural settings, and health contexts [20]. The “non-EPPM” constructs in the online *JH∼PHIRST* survey were derived from our original paper-based Maryland 2005 pilot study [8], which itself was based on validated risk communication theory [15], [21] in the context of an identified set of potential peripheral risk perception influences from emergency preparedness training experiences in local health departments [16].

### Study participants

Four clusters of local health departments from three states in the Midwestern and Eastern U.S. participated in the *JH∼PHIRST* survey. Each region (cluster) had access to the online version of *JH∼PHIRST* via the SurveyMonkey (SurveyMonkey.com, Portland OR) web site for 4 to 6 weeks, and each region's survey results were logged separately. The health departments were responsible for encouraging all of their employees to respond to this survey and individual participation was voluntary. During the survey administration, completion rates by health department were intermittently provided to pre-designated administrative points of contact at the participating agencies within the cluster to encourage agency-wide survey participation.

### Statistical analysis

This survey scale did not include a neutral point, and the option of a “don't know” response may have carried the ambivalence stance. Suspecting that the use of the “don't know” response was not random, a sensitivity analysis was performed to determine how the “don't know” responses could best be incorporated into the analysis approach. Subsequently and prior to analysis, “don't know” responses were assigned the construct-specific (or attitude/belief-specific) median value of the Likert-scale responses. These responses were then dichotomized into categories of ≤5 (‘positive response’ or agreement) versus >5 (‘negative response’ or disagreement). Four scenario-specific categories for the EPPM were also created, based on level of perceived threat and level of perceived efficacy. These categories include: low threat and low efficacy (LT/LE), low threat and high efficacy (LT/HE), high threat and low efficacy (HT/LE), and finally high threat and high efficacy (HT/HE). Using the Likert-scale responses, the ‘threat’ variable was determined as the product of the participant's response to the perceived likelihood of the occurrence of the given public health threat and the perceived severity of the event constructs, while the ‘efficacy’ variable was calculated as the product of the participant's response to their perceived ability to perform their duty (Self Efficacy) and their perceived impact on combating the given public health threat (Response Efficacy) constructs. Low and high categories of perceived threat and efficacy were determined by the median value of each product, respectively.

Pearson chi-square tests were used to compare regions on demographic characteristics and on agreement with the dichotomized questions. Multinomial logistic regression analysis was performed to evaluate relationships between the pandemic flu EPPM categories and demographic factors. In addition, logistic regression analysis was utilized to evaluate relationships between these EPPM categories and the attitude and belief responses to the 16 questions not considered in assigning the EPPM categories. In this analysis the EPPM categories were evaluated as predictors with and without adjustment for demographic characteristics. Missing responses were excluded from the analyses. All analyses were performed using STATA version 10.0 (Stata Corporation College Station, TX) and SAS version 9.1 (SAS Institute, Cary, NC).

## Results

Across the four health department regions, 1835 responses were collected for an overall response rate of 83%. Table 1 describes the composition of the four regions of respondents, and their catchment regional demographics based on U.S. Census data [22]. Most respondents were female (81%), over 40 years of age (72%), had at least a Bachelors' degree (73%), worked in their present organization for at least five years (64%), were in their profession for 10 or more years (58%), perceived having a role in responding to a public health emergency (84%), and had a family member dependent on them (67%). The regions were significantly different on most of these characteristics. The level of non-responses ranged from 1 to 2% across the four scenarios, considering all constructs and potential respondents for a given scenario. Similarly, the level of “don't know” responses ranged from 1.1 to 2.6%. Based on the criteria considered and the sensitivity analysis of how best to incorporate the “don't know” responses as a measure of attitudinal ambivalence in subsequent analyses, imputing these responses with the construct-specific “sample” median.was determined to be a reasonable approach.

**Table 1:** Comparison of population and respondent characteristics by survey region.

	Region 1^a^	Region 2^a^	Region 3^a^	Region 4^a^	p-value^b^	Total
**Catchment (population-weighted)**						
Population	1,727,938	1,133,212	1,073,513	1,055,578		NA
Median Income	$62,075	$44,513	$43,552	$34,842		NA
% with Bachelors degree or higher	32	21	21	15		NA
% minority	15	13	17	5		NA
**Respondent characteristics**						
Number responding	668	354	532	281		1835
Response rate	89	88	82	67		83
% female	88	77	77	78	<0.001	81
% 40+ years of age	72	74	69	77	0.14	72
% w/Bachelors degree or higher	81	72	68	63	<0.001	73
% in present organization 5+ years	68	64	62	58	0.04	64
% in profession 10+ years	62	61	56	51	0.006	58
% perceived having role responding to public health emergencies	86	79	83	85	0.044	84
% with a family member dependent on them	67	69	65	68	0.69	67

a Region 1 represents Minnesota Twin Cities Metropolitan Region. Region 2 represents Northeast Central Ohio Region. Region 3 represents West Central Ohio. Region 4 represents six public health preparedness regions in West Virginia, covering the Eastern, North Central, Central, Western, and Southwestern parts of the state.

b Pearson chi-square analysis.

The overall percent agreement with the 20 pandemic influenza attitude and belief questions ranged from 94% for the perceived need for pre-event preparation and training, the severity of consequences, and the likelihood of being asked to report, to 74% for awareness of role-specific responsibilities (Table S1). Self-reported willingness to respond if required had 92% agreement, and self-reported willingness to respond if asked but not required had 86% agreement. For most of the questions, there was no significant difference in the percent agreement (‘positive’ responses) between regions as shown in Table S1. The three questions with the most inter-regional agreement for percent of positive responses (highest p-value) are the perceived likelihood of being asked to report to duty, self-reported willingness to respond if required, and the ability of the Health Department to provide timely information. Statistically significant inter-regional differences were observed for only three questions: self-reported willingness to respond if asked but not required, awareness of role-specific responsibilities, and skills for role-specific responsibilities. There was also no significant difference (p = 0.74) in the distribution of EPPM categories across the four regions (Table S1).

As a novel approach for understanding willingness to respond to a public health emergency, two relationships with EPPM were considered. The first approach considered whether demographic characteristics from Table 1 were related to the EPPM category assigned to a participant based on their responses to the four constructs noted in the statistical analysis section (Table 2). Region, age, educational level and expected role in emergency response were significant predictors of differences in the multinomial odds ratio (MOR) between EPPM categories. The largest significant MOR (95%CI) is 5.54 (3.36, 9.12), and indicated that the odds favoring being in the high threat/high efficacy EPPM category over the reference (low threat/low efficacy) category were 5.54 times higher for respondents having a perceived expected role in emergency response than for those not perceiving such a role.

**Table 2:** Associations of demographic characteristics with categories of the Extended Parallel Process Model (EPPM) for a pandemic influenza emergency.

	Low Threat, High Efficacy	High Threat, Low Efficacy	High Threat, High Efficacy
	MORa,b (95%CI)	MORa,b (95%CI)	MORa,b (95%CI)
Region			
Region 2 (Region 1 - Reference)	1.08 (0.71–1.65)	1.05 (0.71–1.54)	1.20 (0.84–1.71)
Region 3 (Region 1 - Reference)	1.09 (0.74–1.61)	1.09 (0.76–1.56)	1.43 (1.04–1.97)
Region 4 (Region 1 - Reference)	1.32 (0.83–2.10)	1.21 (0.78–1.90)	1.65 (1.10–2.47)
Gender			
Male (Female - Reference)	0.8 (0.54–1.19)	0.87 (0.61–1.24)	0.54 (0.38–0.76)
Age			
> = 40 years (<40 years - Reference)	1.79 (1.18–2.72)	1.05 (0.74–1.50)	1.45 (1.04–2.03)
Highest Degree			
Bachelors (High school/GED - Reference)	0.81 (0.56–1.19)	1.20 (0.83–1.73)	1.20 (0.85–1.69)
Graduate degree (High school/GED - Reference)	0.96 (0.65–1.44)	1.46 (0.99–2.13)	1.94 (1.38–2.74)
Work duration in organization			
> = 5 years (<5 years - Reference)	1.00 (0.70–1.42)	1.02 (0.74–1.42)	1.22 (0.90–1.66)
Work duration in profession			
> = 10 years (<10 years - Reference)	0.89 (0.62–1.28)	1.06 (0.76–1.49)	1.14 (0.84–1.54)
Expected role in emergency response			
Yes (No - Reference)	1.80 (1.19–2.72)	1.08 (0.76–1.53)	5.54 (3.36–9.12)
Have family member dependent on care			
Yes (No - Reference)	1.10 (0.80–1.50)	0.88 (0.65–1.18)	0.82 (0.62–1.07)

a MOR is the multinomial odds ratio provided in the multinomial logistic regression which compares the odds ratios between this category and the Low Threat/Low Efficacy category as the Reference with respect to a particular characteristic category against its reference category, adjusting for all other characteristics.

b Analysis was based on 1605 participants with available information across all characteristics and questions pertaining to the EPPM categories.

A second set of analyses investigated the ability of EPPM categories to predict responses to the attitude and belief questions, with specific interest in the willingness-to-respond questions. Table 3 shows the results of these analyses and Table 4 shows these results adjusted for the demographic characteristics noted in Table 2. Overall the adjustment for demographic characteristics did not change the relationships of EPPM categories as predictors for any of the attitude/belief questions. The odds of a positive response to a question were significantly higher for the high threat/high efficacy (HT/HE) category than for the low threat/low efficacy (LT/LE) category. For example, the OR for answering positively to the perceived importance of one's role in the agency's overall response for the HT/HE category was 111.08 times larger than for the LT/LE category.

**Table 3:** Associations of categories of the Extended Parallel Process Model (EPPM) with attitudes and beliefs regarding a pandemic influenza emergency (unadjusted for demographic characteristics).

	Low Threat, High Efficacy	High Threat, Low Efficacy	High Threat, High Efficacy
Attitudes and Beliefs	Odds Ratioa,b (95%CI)	Odds Ratioa,b (95%CI)	Odds Ratioa,b (95%CI)
Perceived likelihood of being asked to report to duty	14.19 (4.53–64.44)	3.07 (1.77–5.32)	774.31 (1.57–>999.9)^c^
If required: self-reported willingness to respond	18.14 (5.70–57.66)	2.61 (1.63–4.18)	31.7 (10.00–100.51)
If asked but not required: self-reported willingness to respond	5.73 (3.25–10.12)	1.53 (1.08–2.16)	9.52 (5.52–16.44)
Perceived knowledge about the public health impact	5.39 (3.16–9.22)	1.84 (1.29–2.62)	17.43 (8.80–34.52)
Perceived awareness of role-specific responsibilities	3.76 (2.59–5.45)	1.41 (1.06–1.87)	7.93 (5.45–11.54)
Perceived skills for role-specific responsibilities	7.25 (4.12–12.75)	1.45 (1.05–2.00)	11.29 (6.66–19.13)
Perception of psychological preparedness	4.78 (2.90–7.87)	1.15 (0.84–1.57)	8.64 (5.29–14.12)
Perceived ability to safely get to work	9.65 (4.85–19.21)	1.52 (1.08–2.13)	6.48 (4.11–10.21)
Confidence in personal safety at work	4.89 (3.20–7.47)	1.27 (0.96–1.70)	6.19 (4.31–8.88)
Perception that family is prepared to function in absence	4.19 (2.76–6.37)	1.38 (1.02–1.85)	4.16 (2.98–5.81)
Perceived ability of Health Department to provide timely information	5.08 (2.82–9.16)	2.01 (1.35–2.99)	10.78 (5.75–20.20)
Perceived ability to address public questions	4.74 (3.12–7.19)	1.56 (1.16–2.10)	8.74 (5.80–13.15)
Perception of the importance of one's role in the agency's overall response	13.72 (6.92–27.22)	2.03 (1.47–2.82)	111.08 (27.43–449.92)
Perceived need for pre-event preparation and training	4.98 (2.27–10.97)	3.71 (1.94–7.09)	20.63 (6.46–65.85)
Perceived need for post-event psychological support	1.52 (1.08–2.13)	2.60 (1.81–3.74)	3.60 (2.55–5.08)
Willingness to respond regardless of severity	10.87 (5.65–20.92)	1.79 (1.29–2.49)	11.22 (6.71–18.74)

a The odds ratio compares this category to the Low Threat/Low Efficacy category as the Reference.

b The number of participants included in the analysis for each question was approximately 1680.

c All responses in the High Threat/High Efficacy category were positive. In order to provide an accurate yet reasonable representation of the relationship between this and the Low Threat/Low Efficacy category, a weighted logistic regression analysis (SAS) was performed adding 0.1 to each cell count. The odds ratio and confidence interval indicate that the odds of a positive response to the attitude/belief is exceedingly greater for the High Threat/High Efficacy group than for the Low Threat/Low Efficacy group.

**Table 4:** Associations of categories of the Extended Parallel Process Model with attitudes and beliefs regarding a pandemic influenza emergency (adjusted for demographic characteristics).

	Low Threat, High Efficacy	High Threat, Low Efficacy	High Threat, High Efficacy
Attitudes and Beliefs	Odds Ratioa,b (95%CI)	Odds Ratioa,b (95%CI)	Odds Ratioa,b (95%CI)
Perceived likelihood of being asked to report to duty	20.95 (5.09–86.16)	2.79 (1.60–4.87)	>999.9 (<0.001–>999.9)^c^
If required: self-reported willingness to respond	16.48 (5.16–52.65)	2.39 (1.48–3.87)	41.58 (10.15–170.40)
If asked but not required: self-reported willingness to respond	5.31 (2.93–9.61)	1.43 (1.00–2.04)	8.46 (4.77–15.01)
Perceived knowledge about the public health impact	5.93 (3.31–10.60)	1.73 (1.19–2.52)	14.34 (7.16–28.74)
Perceived awareness of role-specific responsibilities	3.61 (2.43–5.35)	1.37 (1.01–1.84)	6.55 (4.45–9.64)
Perceived skills for role-specific responsibilities	7.71 (4.19–14.20)	1.35 (0.97–1.88)	10.18 (5.94–17.43)
Perceived psychological preparedness	5.02 (2.96–8.52)	1.07 (0.78–1.48)	8.56 (5.12–14.30)
Perceived ability to safely get to work	10.17 (4.90–21.14)	1.43 (1.01–2.03)	5.95 (3.72–9.50)
Confidence in personal safety at work	4.63 (2.99–7.16)	1.25 (0.93–1.69)	6.30 (4.31–9.22)
Perception that family is prepared to function in absence	4.38 (2.81–6.83)	1.45 (1.06–2.00)	4.61 (3.23–6.58)
Perceived ability of Health Department to provide timely information	5.14 (2.78–9.51)	1.99 (1.32–2.98)	11.3 (5.81–21.99)
Perceived ability to address public questions	5.04 (3.23–7.86)	1.52 (1.11–2.07)	8.84 (5.67–13.79)
Perception of the importance of one's role in the agency's overall response	12.80 (6.40–25.60)	1.93 (1.37–2.71)	88.88 (21.86–361.40)
Perceived need for pre-event preparation and training	6.80 (2.70–17.16)	3.83 (1.94–7.56)	19.27 (5.98–62.12)
Perceived need for post-event psychological support	1.52 (1.06–2.17)	2.69 (1.84–3.93)	3.44 (2.39–4.96)
Willingness to respond regardless of severity	10.89 (5.46–21.75)	1.74 (1.24–2.44)	11.00 (6.43–18.84)

a The odd ratios compares this category to the Low Threat/Low Efficacy category as the Reference.

b The number of participants included in the analysis for each question was approximately 1600.

c The Odds Ratio and 95%CI could not be calculated with adjustments for the demographic characteristics because all responses were positive. This odds ratio and CI indicate that the odds of a positive response to the attitude/belief is exceedingly greater for the High Threat/High Efficacy group than for the Low Threat/Low Efficacy group.

A trend previously seen in region-specific analyses (reports to the agencies) and reiterated in Tables 3 and 4 was that the EPPM efficacy dimension tended to have a larger impact on a positive response to an attitude/belief question than the EPPM threat dimension. With respect to the reference (LT/LE) category, for example, the LT/HE category for self-reported willingness to respond if required had a higher OR of a positive response than the HT/LE category. This pattern, observed in 12 of the 16 construct questions, may suggest that the efficacy dimension has a larger impact on positive responses to the attitude and belief questions than the threat dimension.

Three of the 16 questions specifically addressed a respondent's willingness to respond. The OR (95%CI) of answering positively to self-reported willingness to respond if required was 31.7 (10.0, 100.51) times higher for the HT/HE group than for the LT/LE group. The comparable OR (95%CI) for self-reported willingness to respond if asked but not required was 9.52 (5.52, 16.44) and for willingness to respond regardless of severity was 11.22 (6.71, 18.74).

The vast majority (94%) of the respondents believed they will be called upon to respond to duty during an influenza pandemic (Table S1). However, follow up analyses indicate that health department employees who considered their individual roles to be important in the context of overall agency response efforts were, after adjustment for demographic characteristics, 8.45 (95%CI: 6.06, 11.77) times more likely to indicate they would report to duty during a pandemic even if it were severe. Ninety-one percent of the clinical staff felt their job during an influenza pandemic would be important, as compared with 85% among non-clinical staff, while only 73% of the non-clinical staff are aware of their role-specific responsibilities, as compared with 77% in the clinical staff.

## Discussion

Willingness to respond is a critical component of effective public health system readiness and sustainability in emergencies. So far, on a national level, public health readiness and response efforts have focused nearly exclusively on enhancing ability, without specifically attending to willingness issues. Our study results suggest that this response willingness is not to be taken for granted in the public health arena. With some regional variation, overall 16% of the workers in 2006-7 were not willing to “respond to a pandemic flu emergency regardless of its severity”. This number is reassuring in contrast to that reported in previous studies, where higher percentages indicated they would not be willing to respond to an influenza pandemic [8], [10]. However, the workload in public health agencies during a pandemic will be so immense that “all hands on deck” will be required to tackle the resulting challenges, and significant changes in roles and responsibilities will be required. Reported unwillingness to respond by approximately 1 in 6 means that additional efforts are required to increase and sustain the proportion of local health department employees willing to respond. It simultaneously highlights the critical importance of understanding the reasons why some public health employees are unwilling to respond to a pandemic threat.

In this study, we address some of these gaps through systematic application of a behavioral model that addresses cognitive and emotional dynamics of response willingness attitudes. As a theoretical model based on decades of prior research on fear campaigns and health risk messaging, the EPPM describes how a sequence of threat appraisals (perceived severity and susceptibility) and efficacy appraisals (perceived response efficacy and self-efficacy) may influence behavioral responses to messages with fear content.

Using the EPPM, we can see how public health workers' individual degrees of perceived threat (‘concern’) and perceived efficacy (‘confidence’) influence their willingness to respond. Indeed, in line with our basic hypothesis, we have found that individuals who had a perception of high threat and high efficacy – i.e., those who fit a ‘concerned and confident’ profile in the EPPM analysis – had the highest declared self-reported willingness to respond (if required) rates to pandemic flu, which was 31.7 times higher than those fitting a ‘low threat/low efficacy’ EPPM profile.

Of the two basic components of the EPPM – perceived threat and perceived efficacy – the latter proves to be the more significant component in determining willingness in this scenario. Compared to the low threat/low efficacy reference category, low threat/high efficacy increases the willingness to respond if required almost 18-fold, while high threat/low efficacy is associated with less than a 3-fold increase. These results, assessed in 2006-7 following several years of increased awareness and high profile pandemic flu preparedness efforts at federal, state and local agencies, reveal a unique opportunity to induce change.

As any amount of additional assistance will make a difference in response to an influenza pandemic, the first step is to better educate public health workers as to their designated roles during this emergency scenario, and then motivate them with an understanding of why this role makes a difference. If a specific designated role cannot be predetermined, a set of potential roles have to be defined and adequately introduced to all relevant workers, up to a point at which they will feel confident in their ability to perform their duty, and perceive it as important.

Our results also suggest that downplaying the threat of the scenario to ‘calm’ the fears of the workers is not an advisable approach. A sense of threat is an important component in the worker's motivation to prepare for the event and to respond to it. It is important to note that 24% of the respondents did not perceive their work environment as safe, and 15% of the respondents felt they could not safely arrive to work. These issues must be an important component of the workers' training, and some assurance to their personal safety can and should be provided.

Certain limitations to the current study must be acknowledged. First, while we have strived to minimize social desirability bias in the construction and phrasing of the *JH∼PHIRST* instrument content, this survey-based study is not necessarily predictive of actual behavior during an event. Second, the findings from our study of local health department personnel may not necessarily translate to similar findings among responders in other cohorts such as hospital employees, EMS, or police; indeed, this is an area worthy of further comparative research study. Third, the imputation for “don't know” responses may result in narrower confidence intervals for the odds ratio estimates; however, our intent is to provide a relative perspective on willingness to respond and the factors that influence it for a given health department and to identify patterns of attitudes that may influence effectiveness of health departments in emergency situations. Despite these caveats, however, ascertaining local health department workers' dispositions toward fulfilling pandemic flu response expectations nonetheless has value for current local public health agency readiness and response efforts and related training needs assessments.

In a pilot study of local Maryland public health personnel in 2005 [8], we illustrated that risk perception influences peripheral to the actual event – such as perceived importance of one's role in an agency response – can markedly affect willingness-to-respond rates. The results at the time indicated that nearly half of the workers noted they are unlikely to respond to duty during a pandemic emergency. In the US, efforts put into planning, training, exercising and increasing awareness of public health roles in disaster response since the introduction of the Pandemic and All-Hazards Preparedness Act [23], as well as the federal, state, and local resources provided in support of the same, may explain some of the improvement in the willingness to respond rates between the surveys. It should be noted, though, that significant changes in the phrasing of the questions from the pilot study do not allow direct comparison of the responses to the current larger-scale multi-state online survey.

In conclusion, our findings point to the EPPM as a useful framework to inform nuanced assessments of levels of – and gaps in – willingness to respond within the local public health infrastructure. Our data indicate that ‘concerned and confident’ local public health employees are most likely to be willing to respond to an influenza pandemic. This finding may allow public health agencies to design, implement, and evaluate training programs focused on emergency response willingness in health departments.

## Supporting Information

Table S1 Percent positive responses to attitude and belief questions and percent in EPPM categories by region
(0.06 MB DOC)

## References

[1] Osterholm MT (2005). Preparing for the next pandemic.. N Engl J Med.

[2] Levin PJ, Gebbie EN, Qureshi K (2007). Can the health-care system meet the challenge of pandemic flu? Planning, ethical, and workforce considerations.. Public Health Rep.

[3] Anonymous (2004). World is ill-prepared for “inevitable” flu pandemic.. Bull World Health Organ.

[4] Institute of Medicine. Hospital-Based Emergency Care: At the Breaking Point (2007).

[5] Health Resources and Services Administration. Public health workforce study http://bhpr.hrsa.gov/healthworkforce/reports/publichealth/default.htm.

[6] Stein BD, Tanielian TL, Eisenman DP, Keyser DJ, Burnam MA (2004). Emotional and behavioral consequences of bioterrorism: planning a public health response.. Milbank Q.

[7] Dimaggio C, Markenson D, T Loo G, Redlener I (2005). The willingness of U.S. Emergency Medical Technicians to respond to terrorist incidents.. Biosecur Bioterror.

[8] Balicer RD, Omer SB, Barnett DJ, Everly GS (2006). Local public health workers' perceptions toward responding to an influenza pandemic.. BMC Public Health.

[9] Cone DC, Cummings BA (2006). Hospital disaster staffing: if you call, will they come?. Am J Disaster Med.

[10] Irvin CB, Cindrich L, Patterson W, Southall A (2008). Survey of hospital healthcare personnel response during a potential avian influenza pandemic: will they come to work?. Prehosp Disaster Med.

[11] Cowan AE, Ching PL, Clark SJ, Kemper AR (2005). Willingness of private physicians to be involved in smallpox preparedness and response activities.. Biosecur Bioterror.

[12] Katz AR, Nekorchuk DM, Holck PS, Hendrickson LA, Imrie AA (2006). Dentists' preparedness for responding to bioterrorism: A survey of Hawaii dentists.. J Am Dent Assoc.

[13] Qureshi K, Gershon RR, Sherman MF, Straub T, Gebbie E (2005). Health care workers' ability and willingness to report to duty during catastrophic disasters.. J Urban Health.

[14] Gershon RR, Qureshi KA, Stone PW, Pogorzelska M, Silver A (2007). Home health care challenges and avian influenza.. Home Healthcare Management Practice.

[15] Sandman PM, Miller PM, Johnson BB, Weinstein ND (1993). Agency communication, community outrage, and perception of risk: three simulation experiments.. Risk Anal.

[16] Barnett DJ, Balicer RD, Blodgett DW, Everly GS, Omer SB (2005). Applying risk perception theory to public health workforce preparedness training.. J Public Health Manag Pract.

[17] McMahan S, Witte K, Meyer J (1998). The perception of risk messages regarding electromagnetic fields: extending the extended parallel process model to an unknown risk.. Health Commun.

[18] Witte K (1992). Putting the fear back into fear appeals: The extended parallel process model.. Communication Monographs.

[19] Smith RA, Ferrara M, Witte K (2007). Social sides of health risks: stigma and collective efficacy.. Health Commun.

[20] Witte K, Allen M (2000). A meta-analysis of fear appeals: Implications for effective public health campaigns.. Health Education & Behavior.

[21] Slovic P, Fischoff B, Lichtenstein S, Kates RW, Hohenemser C, Casperson J (1985). Characterizing Perceived Risk.. Perilous Progress: Managing the Hazards of Technology.

[22] U.S. Census Bureau, State and County QuickFacts http://quickfacts.census.gov/qfd/index.html.

[23] Pandemic and All-Hazards Preparedness Act (2006). Pub L No.

