# Supplementary material for: Assessment of Local Public Health Workers' Willingness to Respond to Pandemic Influenza through Application of the Extended Parallel Process Model
Source: PLoS One. 2009 Jul 24;4(7):e6365. doi: 10.1371/journal.pone.0006365 (PMC2711331; doi:10.1371/journal.pone.0006365)
Supplement: Table S1 — Percent positive responses to attitude and belief questions and percent in EPPM categories by region (0.06 MB DOC) [file pone.0006365.s001.doc]

**Table S1: Percent positive responses to attitude and belief questions and percent in EPPM categories by region**

|  | **Region 1^a^** | **Region 2^a^** | **Region 3^a^** | **Region 4^a^** | **p-value^b^** | **Total** |
| --- | --- | --- | --- | --- | --- | --- |
| ***Attitudes and Beliefs*** |  |  |  |  |  |  |
| **If required: self-reported willingness-to-respond** | **92** | **92** | **92** | **93** | **0.96** | **92** |
| **If asked but not required: self-reported willingness-to-respond** | **86** | **82** | **87** | **90** | **0.03** | **86** |
| **Willingness-to-respond regardless of severity** | **84** | **81** | **83** | **87** | **0.23** | **84** |
| Perceived likelihood of event occurring ^c^ | 81 | 79 | 75 | 80 | 0.07 | 79 |
| Perceived severity of consequences ^c^ | 94 | 94 | 93 | 96 | 0.51 | 94 |
| Perceived likelihood of being asked to report to duty | 94 | 94 | 93 | 94 | 0.98 | 94 |
| Perceived knowledge about the public health impact | 86 | 85 | 84 | 90 | 0.16 | 86 |
| Perceived awareness of role-specific responsibilities | 73 | 73 | 73 | 82 | 0.03 | 74 |
| Perceived skills for role-specific responsibilities | 82 | 86 | 81 | 89 | 0.02 | 83 |
| Perceived psychological preparedness | 81 | 84 | 84 | 87 | 0.09 | 83 |
| Perceived ability to safely get to work | 83 | 88 | 86 | 86 | 0.24 | 85 |
| Confidence in personal safety at work | 73 | 78 | 77 | 79 | 0.16 | 76 |
| Perceived ability to perform duties (Self Efficacy) ^c^ | 87 | 88 | 87 | 90 | 0.47 | 88 |
| Perception that family is prepared to function in absence | 78 | 79 | 75 | 78 | 0.48 | 77 |
| Health Department's perceived ability to provide timely information | 89 | 88 | 89 | 89 | 0.94 | 89 |
| Perceived ability to address public questions | 76 | 79 | 76 | 83 | 0.14 | 78 |
| Perception of the importance of one's role in the agency's overall response | 84 | 84 | 82 | 87 | 0.46 | 84 |
| Perceived need for pre-event preparation and training | 94 | 94 | 95 | 93 | 0.58 | 94 |
| Perceived need for post-event psychological support | 83 | 78 | 78 | 81 | 0.19 | 80 |
| Perceived high impact of one's response (Response Efficacy) ^c^ | 88 | 84 | 86 | 88 | 0.42 | 86 |

^a^ Region 1 represents Minnesota Twin Cities Metropolitan Region . Region 2 represents Northeast Central Ohio Region,. Region 3 represents West Central Ohio. Region 4 represents six public health preparedness regions in West Virginia, covering the Eastern, North Central, Central, Western, and Southwestern parts of the state.

^b^ Pearson chi-square analysis

^c^ There are 4 constructs used to create the EPPM categories. Perceived likelihood of event occurring and perceived severity of consequence define the threat dimension of EPPM. Perceived ability to perform duties (Self Efficacy) and the perceived high impact of one’s response (Response Efficacy) define the efficacy dimension of EPPM.
